# Exosomes‐mediated crosstalk between glioma and immune cells in the tumor microenvironment

**DOI:** 10.1111/cns.14239

**Published:** 2023-05-11

**Authors:** Xu Guo, Rui Sui, Haozhe Piao

**Affiliations:** ^1^ Department of Neurosurgery Cancer Hospital of China Medical University, Liaoning Cancer Hospital & Institute Shenyang China; ^2^ Department of Neurosurgery Cancer Hospital of Dalian University of Technology (Liaoning Cancer Hospital & Institute) Shenyang China

**Keywords:** exosomes, glioma, immune cells, immunotherapy, tumor microenvironment

## Abstract

Gliomas are the most common primary malignant tumors in the central nervous system. However, conventional treatments, such as surgical resection and postoperative combined chemo‐ and radio‐therapy, are ineffective in improving patients' long‐term survival. The tumor microenvironment (TME) consists of stromal cells, tumor components, and innate and acquired immune cells, and these cells, along with the extracellular matrix, regulate and communicate intercellularly to promote TME formation. The immune microenvironment plays a vital role in the development of glioma. Exosomes, which are extracellular vesicles (EVs), facilitate intercellular communication and regulation within the TME. Tumor cells can release exosomes to transmit messages, induce macrophage polarization, and inhibit immune cell activity, ultimately promoting metastasis and immune evasion. Moreover, immune cells can regulate tumorigenesis and progression through exosomes. This review summarized the biological properties of exosomes and their effects on the tumor microenvironment and provides an overview of the interactions between glioma cells and immune cells.

## Graphical abstract

The tumor immune microenvironment contains parenchyma tumor cells and immune cells. By releasing exosomes, tumor cells can transmit messages, induce macrophage polarization, and inhibit immune cell activity, thus promoting metastasis and immune escape. Immune cells can also regulate glioma cells genesis and progression through exosomes.

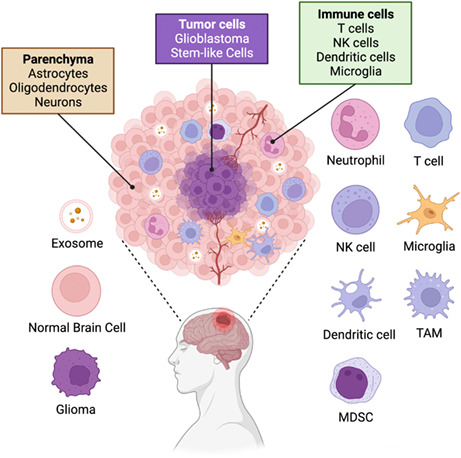

## 1 INTRODUCTION

Gliomas are malignant tumors originating from the central nervous system and are characterized clinically by high morbidity, mortality, high infiltrating, and poor prognosis.^1^, ^2^ The main treatments for glioma are direct surgical resection, radiotherapy, and temozolomide chemotherapy.^3^, ^4^, ^5^, ^6^, ^7^ Gliomas are classified as astrocytic, oligodendroglial, and ventricular canal tumors based on their malignancy and phenotype.^8^, ^9^, ^10^ Diffuse gliomas can be further typed as astrocytic, oligodendroglial, or rare mixed oligodendroglial‐astrocytic of World Health Organization (WHO) grade II (low grade), III (anaplastic), or IV (glioblastoma). According to the WHO histological classification, gliomas are classified as WHO grade I–IV, with glioblastoma, a WHO grade IV glioma, accounting for approximately 45% of malignant brain tumors.^11^, ^12^ The pathogenesis of glioma remains unclear, with tumorigenesis resulting from a combination of environmental and genetic factors.

The tumor microenvironment (TME) consists of tumor cells and surrounding components.^13^, ^14^ It includes innate and adaptive immune cells (T lymphocytes and B lymphocytes), mesenchymal fibroblasts, and vascular and lymphatic vessel networks. Various chemokines secreted through autocrine or paracrine make up the TME.^15^, ^16^, ^17^ Alterations in the microenvironment affect tumorigenesis and progression. The immune cell is fundamental in determining the fate of cancer and its invasiveness and metastatic capacity.^18^, ^19^ The clinical outcome of cancer patients is interrelated to the composition of immune cells that infiltrate tumors.^20^, ^21^

Exosomes are extracellular vesicles of 30–150 nm in diameter released in the form of exocytosis. The intake of exosomes relies on the endocytic system after fusion with the cell membrane.^22^, ^23^, ^24^ Exosome contains various bioactive components,^25^, ^26^, ^27^, ^28^ and most cells (e.g., mast cells, tumor cells, dendritic cells, neuron‐shaped glial cells) can generate exosomes.^29^, ^30^, ^31^ The outer membrane of exosomes has the property of a cytosolic phospholipid bilayer, effectively maintaining the activity and preserving stability in various body fluids.^32^, ^33^ The exosomes are abundant in TME and function as the most critical information exchange tool between tumor cells and TME.^34^, ^35^, ^36^, ^37^, ^38^

Exosomes derived from tumor cells promote immune escape by directly inhibiting immune cells or regulating the expression of related cytokines.^39^, ^40^ Moreover, immune cells in the TME influence tumor cell growth, metastasis, and drug resistance by secreting exosomes.^41^, ^42^, ^43^ This article reviewed the regulation of exosomes in the glioma TME and their role in the malignant progression of glioma.

## 2 OVERVIEW OF EXOSOMES

### 2.1 Mechanism of exosome formation

Exosomes are generated in normal physiological conditions or response to external environmental stimuli. They originate from intracellular invaginations, where the cell membrane forms multiple vesicles. These vesicles fuse to form early intracellular vesicles, which further mature and bud inward to form luminal vesicles or intraluminal vesicles (ILVs).^44^, ^45^, ^46^ ILVs‐rich intracellular bodies are called multi‐vesicular bodies (MVBs). Upon fusion with lysosomes intracellularly, MVBs are degraded, while the other part is secreted outward to form exosomes under the regulation of Rab enzymes of the GTPase family.^47^, ^48^, ^49^ Exosomes are extracellular vesicles with a diameter of 30–150 nm generated by almost all cell types.^50^, ^51^ Exosomes consist of a lipid bilayer containing transmembrane proteins and wrap around cytoplasmic proteins, lipids, or nucleic acids.^52^, ^53^ Exosomes can regulate cellular communication by transporting specific exosomal contents.^54^, ^55^ Exosomes are closely associated with normal physiological homeostasis and various diseases, including cancer. Exosomes are essential mediators of tumorigenesis, proliferation, angiogenesis, and distant metastasis.^56^, ^57^ Currently, the extraction of exosomes has become commercially available. The advantages and disadvantages of different methods are presented in Table 1.

**TABLE 1:** The separation techniques for exosomes.

Detection methods	Advantages	Disadvantages
Ultracentrifugation	The obtained exosomes are not contaminated by the separation reagent, and the number of separations is large, and the processed sample is small	The instrument is expensive, the sample volume is large, the time consumption is long, and protein contamination still exists when exosomes are observed by electron microscopy
Sucrose Density Gradient Centrifugation	High purity of exosomes	The preliminary preparation is complicated, time‐consuming, and cannot completely separate exosomes from proteins
Polyethylene Glycol (PEG)	Simple operation, no special equipment required, more economical, and high yield of exosomes	Precipitation of some non‐exosome hydrophobic substances resulting in insufficient exosome purity
Kit method	Simple, less time‐consuming, and higher yield of exosomes	The obtained exosome precipitate contains many impurities, and samples from different sources need to be extracted using different kits, and the kits are more expensive
SEC	High purity of exosomes enables the isolation of structurally intact and functionally active vesicles	Special equipment required
Ultrafiltration	Rapid separation of exosomes of different sizes with high capture efficiency	Filters can easily become clogged with vesicles and other macromolecular substances, causing the membrane to become overstressed and broken
Separation method is based on surface component affinity	Higher recovery and less impurity protein	Based on special protein adsorption
ACE separation method	The ability to simplify the exosome extraction and recovery process, significantly reducing processing steps and time‐consuming	Requires an AC electric field to be applied
Microfluidic chip method	Simple operation and high capture rate	Requires nanofilters

### 2.2 Exosomes functions

Exosomes are widely distributed in the body fluid of tumor patients after being released.^58^, ^59^ They can enter and deliver biologically active substances to target cells, affecting gene expression, protein synthesis, and other processes that ultimately regulate the target cells' function.^60^, ^61^ Exosomal surface proteins bind directly to target cell receptors and stimulate signaling pathways, and they can also fuse with target cell membranes and deliver functional proteins, miRNA, and other biomolecules.^22^, ^62^ The mRNA of exosomes can translate corresponding proteins, and miRNA and siRNA regulate target cells by affecting the expression of related genes.^63^, ^64^ Phagocytose exosomes can either be re‐released in target cells or degraded by the lysosomal pathway.^65^, ^66^

The exosomal function is closely related to their cellular origin and the protein and RNA they contain. Exosomes from different sources have different purposes at different physiological and pathological stages.^67^, ^68^ Exosomes can regulate physiological activities and maintain intracellular homeostasis, support the body's immune tolerance, and participate in normal physiological processes, and pathological processes.^69^, ^70^ Tumor cell‐derived exosomes contain antigens, genetics, and other biologically active substances that are vital in tumorigenesis, proliferation, invasion, and metastasis.^71^ Proteins, miRNAs, and even DNA in tumor‐derived exosomes are potential markers for non‐invasive diagnosis.^72^, ^73^ As natural carriers, the phospholipid bilayer structure of exosomes protects the stability of proteins, miRNA, and other biologically active substances. Exosomes have similar biological activities as parental cells, are widely distributed, exist for a long time in the body, and have the advantages of a long half‐life and natural nontoxicity. Due to their nanoscale structure, exosomes can evade phagocytosis while freely shuttling between cells and matrix, with solid penetration ability and low immunogenicity.^74^ Exosomes can also serve as a means of delivering drugs and miRNAs for tumor therapy. Tumor cell‐derived exosomes are also potential for tumor vaccine development.^75^

### 2.3 Immune system cells‐derived exosomes (IEXs)

IEXs have a comprehensive spectrum of functions in the immune system. IEXs regulate multiple immune signaling pathways, including modulation, antigen presentation, antitumor immunity, and immune system suppression. Dendritic cell‐derived exosomes (DEX) can enhance antitumor immunity and activate specific T cells in the direct pathway by expressing the MHCII‐peptide complexes, costimulatory molecules, and binding to T‐cell receptors. In the indirect pathway, DEXs deliver the MHC II‐peptide complex to other DCs, a process named MHC‐dressing. Exosomes can transfer antigenic peptides from activated to inactivated DCs, which increases the number of MHCII‐peptide complexes on the surface of DCs to activate the T cells. Macrophage‐derived exosomes contain over 5100 proteins, and exosomal miR‐21‐5p and miR‐155‐5p secreted from nonclassical macrophages (M2) regulate tumor cells' migration, proliferation, invasion, and angiogenesis. Neutrophils‐derived exosomes have been shown to have unique protein profiles that change in activated and non‐activated situations. Immune cells generate exosomes with their properties that create an optimal microenvironment for function by activating other immune cells, inhibiting immune responses, and participating in the licensing phenomenon of APCs. In the following, we will discuss the characteristics of the exosomes of each cell involved in the glioma interaction with the immune system.

## 3 TUMOR MICROENVIRONMENT

**FIGURE 1:**
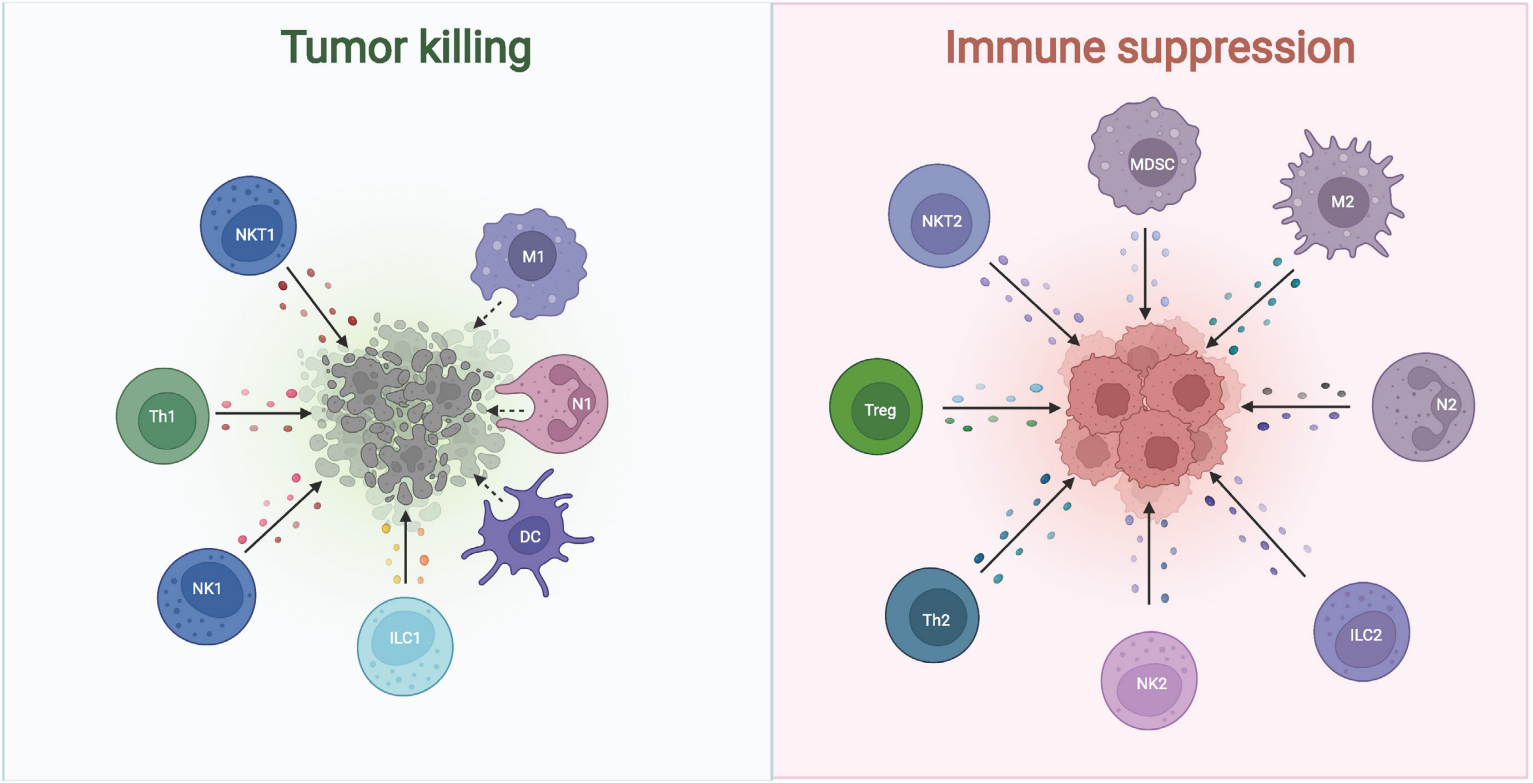
The primary cells involved in the tumor immune microenvironment. In the tumor immune microenvironment, some cells (NKT1, M1, N1, DC, ILC1, NK1, and Th1) directly kill tumor cells, while others (MDSC, M2, N2, ILC2, NK2, Th2, and Treg) cause immunosuppression. N1, N1‐polarized neutrophils; N2, N2‐polarized neutrophils.

Tumor cells typically invade normal tissues and establish a tumor microenvironment (TME) consisting of immune cells, stromal cells, vascular endothelial cells, and extracellular matrix (ECM).^76^, ^77^, ^78^, ^79^ Tumor cells can recruit and activate these components to form a tumor‐inhibiting inflammatory microenvironment, which can prevent tumor progression during the early stages of colonization or proliferation^80^, ^81^ (Figure 1). However, persistent tumor antigen stimulation and immune activation can lead to a depleted or remodeled state of the effector cells in the microenvironment, resulting in an immunosuppressive microenvironment that promotes malignant tumor phenotypes^82^, ^83^, ^84^ (Figure 1). Immunotherapeutic strategies targeted at the TME can stimulate or restore the innate tumor‐suppressive capacity of the immune system, create a favorable immune microenvironment, and produce a comprehensive response.^85^, ^86^, ^87^

**FIGURE 2:**
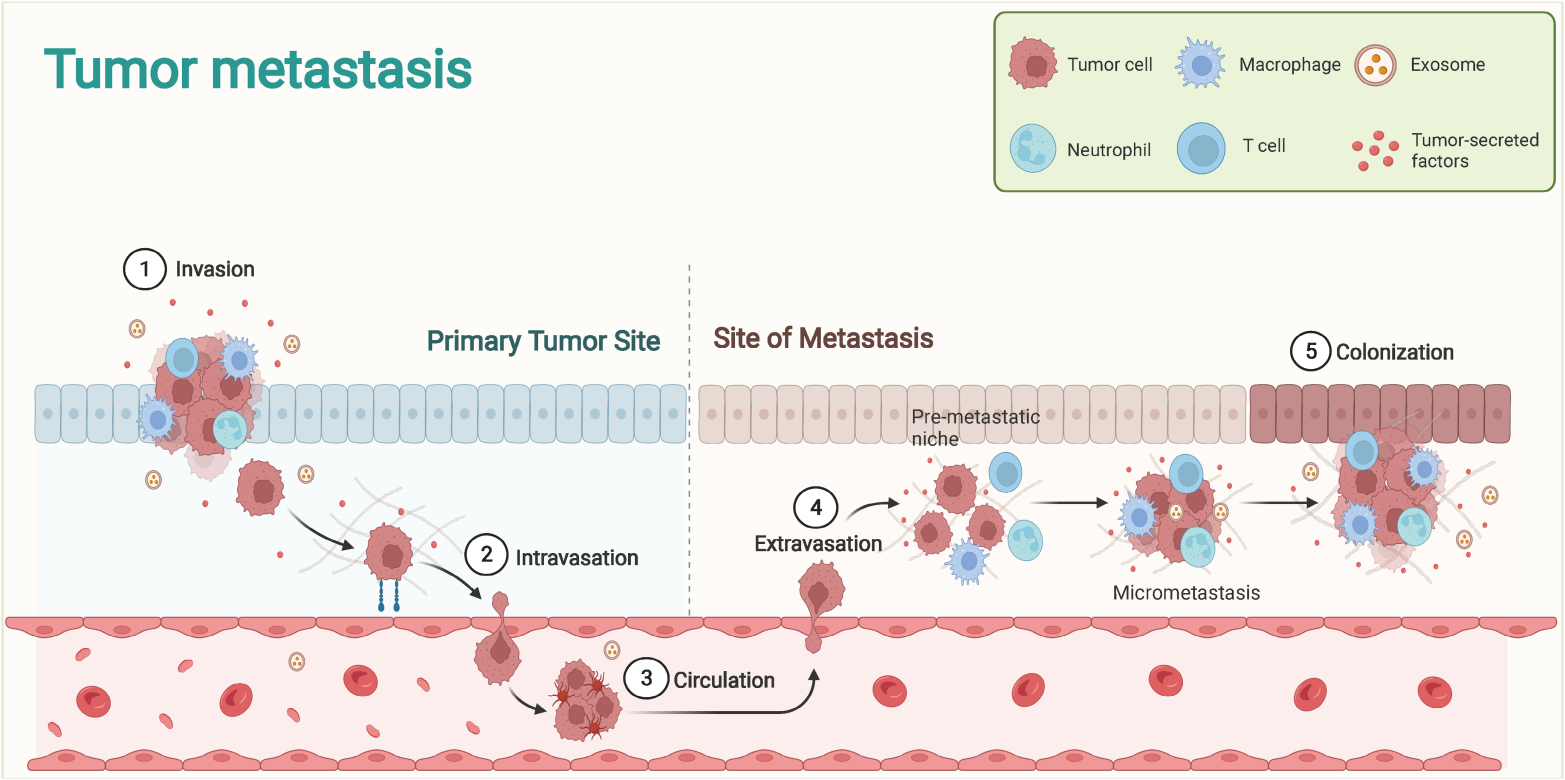
The primary process of tumor metastasis. Tumor metastasis mainly includes invasion, intravasation, circulation, extravasation, and colonization. It also provides for the involvement of macrophages, neutrophils, and T cells.

Tumor metastasis is the primary cause of mortality. The pre‐metastatic microenvironment of tumors refers explicitly to the microenvironment in which the primary tumor focus is prepared for distant dissemination and colonization of tumor cells (Figure 2). The pre‐metastatic microenvironment includes tumor‐derived secretory factors, extracellular vesicles, macrophages, neutrophils, and T cells. With the manipulation of the primary tumor, multiple changes occur at distant sites, including the formation of PMN, inflammation, immunosuppression, angiogenesis/vascular permeability, organophilic, reprogramming, and lymphangiogenesis.

The glioma microenvironment refers to the internal and external environment closely associated with glioma development, proliferation, and metastasis, including the structure, function, and metabolism of the tumor‐host tissue and the intrinsic environment of glioma cells.^88^, ^89^, ^90^ Glioma cells require large amounts of nutrients to meet their metabolic needs. The plasticity of glioma metabolism allows them to adapt to a depleted or changing nutritional environment, reshaping the tumor immune microenvironment.^91^, ^92^, ^93^ The tumor‐promoting effect of glioma‐associated microglia (GAMs), macrophages, regulatory T cells (Treg), and the inactivation of natural killer (NK) cells in TME can reduce the antitumor effect and are closely related to the formation of glioma immunosuppressive microenvironment.^88^, ^94^ In addition to immunosuppressive factors, the TME accumulates metabolites from tumor proliferation, such as adenosine and lactate.^95^, ^96^ Hypoxic TME increases the release of ATP and AMP. The nucleotidases CD39 and CD73 catalyze the conversion of ATP to AMP and AMP to adenosine extracellularly, resulting in a significant increase in adenosine levels in immune regulation.^97^, ^98^

## 4 EXOSOME‐MEDIATED INTERACTION IN GLIOMA

### 4.1 Interaction between glioma and immune cells in the TME

Glioma is a primary malignant tumor in the central nervous system (CNS), characterized by aggressive growth and difficulty in clinical cure. These features may be related to the low immunogenicity of glioma cells and the tumor immunosuppressive microenvironment.^99^ Immune cells in the TME compete with glioma cells for nutrients, and the metabolites produced during this competition can affect immune cell differentiation and function. Glioma cells can influence the process of immune cells by secreting exosomes, and promote their malignant progression (Table 2). Similarly, immune cells can influence glioma progression by delivering substances to glioma cells through exosomes (Table 3).

**TABLE 2:** Overview of glioma cell‐derived exosome cargos and their biological effects in TME.

Donor cell	Exosomal cargos	Receiving cell	Biological effects	References
Glioma cells	/	Monocytes	Suppress T‐cell immune responses	^107^
Glioma cells	miR‐10a/miR‐21/RORA/PTEN	MDSCs	Induce MDSCs expansion and activation	^108^
Glioma cells	miR‐29a/miR‐92a /HBP1/PRKRA1A	MDSCs	Activate the proliferation of MDSCs	^150^
Glioma cells	SDF‐1α/CXCR4	MDSCs	Induces MDSC recruitment	^109^
Glioma cells	miR‐1246/DUSP/ERK	MDSCs	Drive differentiation and activation of MDSCs	^151^
Glioma cells	miR‐1298‐5p/SETD7 /MSH2	MDSCs	Promote immunosuppression of MDSCs	^110^
Glioma cells	miR‐1983/TLR7/MyD88‐IRF5/IRF7/IFN‐β	NK cells	Stimulate NK cell responses	^114^
GL26 cells	IFN‐γ and Granzyme B	CD8^+^ cells	Inhibit cell growth	^121^
GSCs	/	CD4^+^ T	Promote cell growth	^107^
Glioma cells	CD73	T cells	Promote clonal proliferation of T cells	^122^
GBM cells	miR‐451/miR‐21/c‐Myc	Microglia	Inhibit cell growth	^136^
GBM cells	miR‐214‐5p/CXCR5	Microglia	Regulate the inflammatory response of microglia	^137^
Glioma cells	miR‐1246/TERF2IP/STAT3/NF‐κB	Macrophage	Promote M2 macrophage polarization	^138^
GBM cells	Arginase‐1	Macrophage	Promote M2 macrophage polarization	^139^
Glioma cells	IL‐6‐pSTAT3‐miR‐155‐3p‐autophagy‐pSTAT3	Macrophage	Promote M2 macrophage polarization	^141^
Glioma cells	Circ‐NEIL3/HECTD4/IGF2BP3	TAMs	Confer immunosuppressive properties on TAMs	^143^

**TABLE 3:** Overview of immune cell‐derived exosome cargos and their biological effects in TME.

Donor cell	Exosomal cargos	Receiving cell	Biological effects	References
DCs	c‐Cbl/PI3K/Akt/ERK	CD4^+^ and CD8^+^ T cells	Promote cell proliferation and inhibit tumor growth	^102^
DCs	LGALS9	T cells	Promotes DCs tumor antigen‐presenting activity and durable antitumor immunity	^103^
NK cells	IL‐15	Glioma cells	Inhibits the growth of glioblastoma	^152^
Neutrophils	DOX	Glioma cells	Inhibits the growth of glioma	^128^
TAMs	miR‐27a‐3p/miR‐22‐3p/miR‐221‐3p	GSCs	Induction of GSC proneuromesenchymal transition	^153^
M2 microglia	miR‐7239‐3p/BMAL1	Glioma cells	Promote glioma proliferation and migration	^140^
M2 macrophages	miR‐15a/miR‐92/CCND1/RAP1B	Glioma cells	Inhibit tumor cell migration and invasion	^154^
TAMs	Lnc‐TAL/ENO1/p38/MAPK	M2 microglia	Lead to chemotherapy resistance	^155^

### 4.2 Dendritic cells (DCs)

Studies have shown that dendritic cell‐derived exosomes (DEX) can enhance antitumor immunity and activate specific T cells to combat tumor cells. Using methods to upregulate and downregulate exosome production by immune cells is a novel way to regulate immunity against tumors and infected cells, as well as immune reactions in some autoimmune and allergic diseases. DCs are the cells in the T cell‐mediated immune response to cancer within the organism. DCs and their precursors in tumors can be recruited and respond to several molecular signals, including cell death, inactivation, and successful maturation in the TME.^100^ Immature DCs cannot initiate T‐cell responses to tumors and may induce immune tolerance, while mature DCs can migrate to tumor‐draining lymph nodes to create T‐cell responses, recruit T cells into the TME, and produce immunostimulatory cytokines to regulate the TME.^101^

Research suggests that chaperone‐rich cell lysates (CRCLs) may play an essential role in developing antitumor vaccines. DCs from CRCLs loaded with GL261‐derived glioma cells can significantly prolong the survival of tumor‐bearing mice and inhibit tumor growth in vivo by secreting exosomes. The CRCL‐GL261‐DCs promote cell proliferation and cytotoxic T lymphocyte (CTL) activity of CD4^+^ and CD8^+^ T cells in vitro. Mechanism findings suggest that CRCL‐GL261‐DCs can negatively regulate Casitas B cell line lymphoma (Cbl)‐b and c‐Cbl signaling to lead to activation of phosphatidylinositol 3‐kinase (PI3K)/Akt and extracellular signaling‐regulatory kinase (ERK) signaling in T cells.^102^ Cerebrospinal fluid (CSF) exosomes from GBM patients contain the unique protein‐LGALS9 ligand, which binds to the TIM3 receptor of DCs in CSF and further interferes DCs recognition, inhibits the presentation of antigen, resulting in the deactivation of cytotoxic T cell‐mediated antitumor immune responses. Blockade of the tumor secretory exosome LGALS9 in a mouse GBM tumor model significantly promoted DCs antigen presentation activity and durable antitumor immunity.^103^ Targeting LGALS9 may be an effective therapeutic tool for GBM.

### 4.3 Myeloid‐derived suppressor cells (MDSCs)

Myeloid‐derived suppressor cells protect tumor cells from immune attack by negatively regulating the immune response, depleting essential amino acids like arginine and cysteine, necessary for T cell activation, and the production of reactive oxygen and nitrogen species that repress T cell function.^104^ MDSCs also induce immune tolerance by recruiting regulatory T cells and Th17 cells through cytokine secretion such as TGF‐β, IL‐10, IFN‐γ, and upregulating ligands like CD86.^100^ Additionally, MDSCs suppress innate immunity by inducing macrophage polarization towards the M2 phenotype and inhibiting NK cell‐mediated cytotoxic effects.^105^ Apart from immunosuppressive functions, MDSCs upregulate vascular endothelial growth factors promoting angiogenesis and tumor growth acceleration.^106^ Stimulated PBMCs from healthy donors with anti‐CD3, anti‐CD28, and IL‐2 with glioma stem cell‐derived exosomes significantly inhibited T cell activation, proliferation, and Th1 cytokine production but enhanced purified CD4^+^ T cell proliferation without affecting cell viability.^107^ Glioma‐derived exosomes repress T‐cell immune responses by acting on monocyte maturation rather than interacting directly with T cells. Hypoxia‐induced glioma‐derived exosomes can more effectively induce MDSCs than normoxia‐induced exosomes by promoting the expression of miR‐10a and miR‐21, targeting RORA, and PTEN.^108^ Additionally, hypoxia‐induced glioma cells can stimulate functional MDSC differentiation by transferring exosomal miR‐92a, activating the proliferative ability of MDSCs by targeting HBP1 and PRKAR1A. Glioma cell line‐derived exosomes, BATF2‐Exo, can inhibit MDSC chemotaxis by inhibiting intracellular SDF‐1α, while exosomal miR‐1246 activates MDSC differentiation and activation via the DUSP3/ERK pathway.^109^ Furthermore, the upregulation of POU5F1 and hnRNPA1 enhances miR‐1246 transcription and packaging. Suppression of hypoxia‐driven exosomal miR‐1246 expression in glioma cells and PD‐L1 expression in MDSCs is a promising new approach for treating glioma. Finally, enrichment of miR‐1298‐5p in CSF exosomes significantly inhibits glioma progression by promoting the immunosuppressive effects of MDSCs and glioma through targeting SETD7 and MSH2.^110^

### 4.4 Natural killer (NK) cells

NK cells represent a significant subset of lymphocytes in innate immunity. They enhance cytotoxicity in the immune response by releasing various cytokines, including perforin, interferon‐γ (IFN‐γ), tumor necrosis factor (TNF), granulocyte‐macrophage colony‐stimulating factor (GM‐CSF), and macrophage inflammatory protein‐1 (MIP‐1).^111^, ^112^ Interleukin‐15 (IL‐15) significantly increases the potential of NK cell‐derived exosomes (NK‐Exo) for immunotherapy. NK‐Exo containing IL‐15 (NK‐Exo‐IL‐15) demonstrated greater cytolytic activity against glioblastoma and significantly increased NK cell cytotoxicity. Neither NK‐Exo nor NK‐Exo‐IL‐15 was toxic to normal cells or mice. NK‐Exo‐IL‐15 inhibited the proliferation of mouse glioblastoma xenografts compared to NK‐Exo.^113^ Glioma‐released exosome‐derived miR‐1983 plays a vital role in the innate antiglioma NK‐mediated circuit regulated by galectin‐1 (Gal‐1). As an endogenous TLR7 ligand, miR‐1983 activates TLR7 in pDC and cDC via the 5′‐UGUUU‐3′ motif at its 3′ end and further activates downstream signaling, stimulating IFN‐β secretion via MyD88‐IRF5/IRF7, and ultimately eradicating gliomas through stimulation of NK cells.^114^

### 4.5 T cells and B cells

Lymphocytes have a complex role in tumor immune escape and inhibition of tumor growth.^115^, ^116^ CD4^+^ T cells and CD8^+^ T cells can hinder tumor growth and metastasis through specific immune responses.^117^, ^118^ However, regulatory B cells (Bregs) utilize immune resistance through Tregs and can promote tumor immune escape.^119^, ^120^ GL26 cell‐derived exosomes significantly suppressed the percentage of CD8^+^ T cells in splenocytes by inhibiting the release of IFN‐γ and granzyme.^121^ Glioma stem cell (GSC)‐derived exosomes function as mediators of intercellular communication and promote tumor immune escape by inhibiting T cell activation, proliferation, and Th1 cytokine production while enhancing the proliferation of purified CD4^+^ T cells. Furthermore, glioma‐derived exosomes in PBMC directly promote the production of IL‐10 and arginase 1 by unstimulated CD14^+^ monocytes and down‐regulate HLA‐DR, producing a phenotype similar to monocyte myeloid‐derived suppressor cells (Mo MDSCs).^107^ The concentration of exosome‐derived CD73^+^ in tumor‐derived extracellular vesicles (TDEVs) was significantly higher in the body fluids of GBM patients. In vitro, results showed that T cells could take up CD73^+^ TDEVs released from GBM cells. In vivo, defects in exosome synthesis and CD73 expression significantly inhibited tumor growth in GBM‐bearing mice and restored clonal proliferation of T cells in central and peripheral regions.^122^

### 4.6 Macrophage

The macrophage‐derived exosomes contain over 5100 proteins, with their composition changing upon activation. Activated macrophage exosomes promote inflammation by triggering the NLRP3 receptor‐dependent inflammasome, TOLL‐like receptors (TLR), and TNF‐related signaling pathways. Additionally, macrophage‐derived exosomes inhibit the expression of β1 integrins, thus inhibiting endothelial and tumor migration. Exosomes generated from nonclassical macrophages (M2) containing high levels of miR‐21‐5p and miR‐155‐5p molecules regulate tumor migration, proliferation, invasion, and angiogenesis. The miR‐21‐5p enhances the proliferation and drug resistance of tumor cells by targeting PTEN, P21, PCD, and apoptotic protease activating factor 1 (APAF1). On the other hand, miR‐130b‐3p expression level increases in MB patients' plasma exosomes, inhibiting the proliferative ability of tumor cells via inhibiting the SIK1 and p53 signaling pathways. The expression levels of miR‐101‐3p and miR‐423‐5p are significantly higher in MB patients' plasma exosomes than in healthy controls.^123^ Moreover, exosomes carrying miR‐101‐3p and miR‐423‐5p suppress tumor cell proliferation, migration, and invasion and enhance cell apoptosis, which could be tumor‐suppressive by targeting the FoxP4 gene. Furthermore, miR‐101‐3p can target EZH2 to increase its tumor‐suppressive effect.^124^

### 4.7 Neutrophils

Neutrophils are the most abundant type of leukocytes and play a crucial role in the inflammatory response's progression.^125^ Their exosomes are essential for innate immunity, as neutrophils are among the first cells present at the site of inflammation. However, whether neutrophils exhibit pro‐ or antitumor properties in a microenvironmentally relevant manner depends on multiple cytokine receptors on their surface, enabling them to respond to various signals.^126^, ^127^ Activated neutrophil‐derived exosomes can alter airway smooth muscle cells' proliferation and biological properties, suggesting their role in airway structural changes in asthma. A bio‐inspired neutrophil‐exosome (NEs‐Exos) system has been identified for delivering doxorubicin drugs for glioma treatment. In vivo experiments have shown that drug‐loaded NEs‐Exos can penetrate the BBB and migrate to the brain. Moreover, NEs‐Exos can chemically respond to inflammatory stimuli and target infiltrating tumor cells in inflamed brain tumors. Intravenous injection of NEs‐Exos/DOX effectively inhibits tumor growth and prolongs survival in a glioma mouse model.^128^

### 4.8 Tumor‐associated macrophages (TAMs)

Tumor‐associated macrophages play a crucial role in tumor growth, invasion, and metastasis. These inflammatory immune cells have two forms of activation, the M1 type and the M2 type.^129^ M1 TAMs have antitumor properties and effectively identify and destroy cancer cells through phagocytosis and cytotoxicity.^130^, ^131^ They perform key functions, such as secreting toxic intermediates and various inflammatory factors, activating Th1 cells, participating in the Th1 immune response, and having proinflammatory and antitumor effects.^132^, ^133^ In contrast, M2 TAMs can promote tumor progression by secreting cytokines that regulate tumor growth and angiogenesis and reduce patient survival.^134^, ^135^ The expression of miR‐451/miR‐21 is significantly increased in GBM‐derived exosomes, leading to the malignant progression of GBM by targeting c‐Myc mRNA expression.^136^ Additionally, exosomal miR‐214‐5p derived from GBM can regulate the inflammatory response of microglia via targeting CXCR5.^137^ Studies have shown that HGDEs, unlike GDEs, significantly promote glioma proliferation, migration, and invasion by inducing M2 macrophage polarization. MiR‐1246 enriched in the CSF of GBM patients can inhibit M2 macrophage polarization by targeting TERF2IP.^138^

TAMs reprogrammed by GBex produce immunosuppressive and tumor growth‐promoting proteins, including Arginase‐1 that promotes glioblastoma growth. However, the pro‐growth effect of Arginase‐1 carried by TAM‐derived exosomes can be reversed by the selective Arginase‐1 inhibitor nor‐NOHA.^139^ MiR‐7239‐3p in M2 microglia exosomes promotes glioma proliferation and migration, while miR‐92 inhibits tumor cell migration and invasion by targeting CCND1 or RAP1B and regulating the PI3K/AKT/mTOR signaling pathway.^140^ H‐GDEs promote autophagy and M2‐like macrophage polarization, thereby promoting glioma progression through the IL‐6‐pSTAT3‐miR‐155‐3p‐autophagy‐pSTAT3 positive feedback loop.^141^ Additionally, the miRNA‐124 delivered by HEK293T‐derived EV exerted synergistic antitumor effects by inhibiting the growth of human GBM cells and suppressing M2 microglia polarization.^142^ Finally, circ‐NEIL3, upregulated in glioma tissue and stabilized by hnRNPA2B1, can promote tumorigenesis and oncogenic progression by blocking HECTD4‐mediated ubiquitination and by driving macrophage infiltration into the TME.^143^

## 5 PROSPECT AND CONCLUSION

The significance of TME in tumor progression is increasingly recognized, as it actively participates in the process of tumor advancement. Tumor cells in TME account for about 30% of the population. Immune cells within the tumor have been found to play either a tumor‐suppressing or promoting role, while some have a bidirectional regulatory role.^144^, ^145^ The functions of both tumor cells and immune cells in the TME are reciprocal, as the behavior of tumor cells is closely linked to tumorigenesis and progression, which then influences the biological behavior of stromal cells in the TME. Immunotherapy strategies have shown positive effects and potential cures for advanced high‐grade tumor patients; however, TME evolves dynamically through compensatory feedback mechanisms, blocking immunotherapeutic effects, generating drug resistance, and even tumor progression.^77^, ^146^, ^147^

**FIGURE 3:**
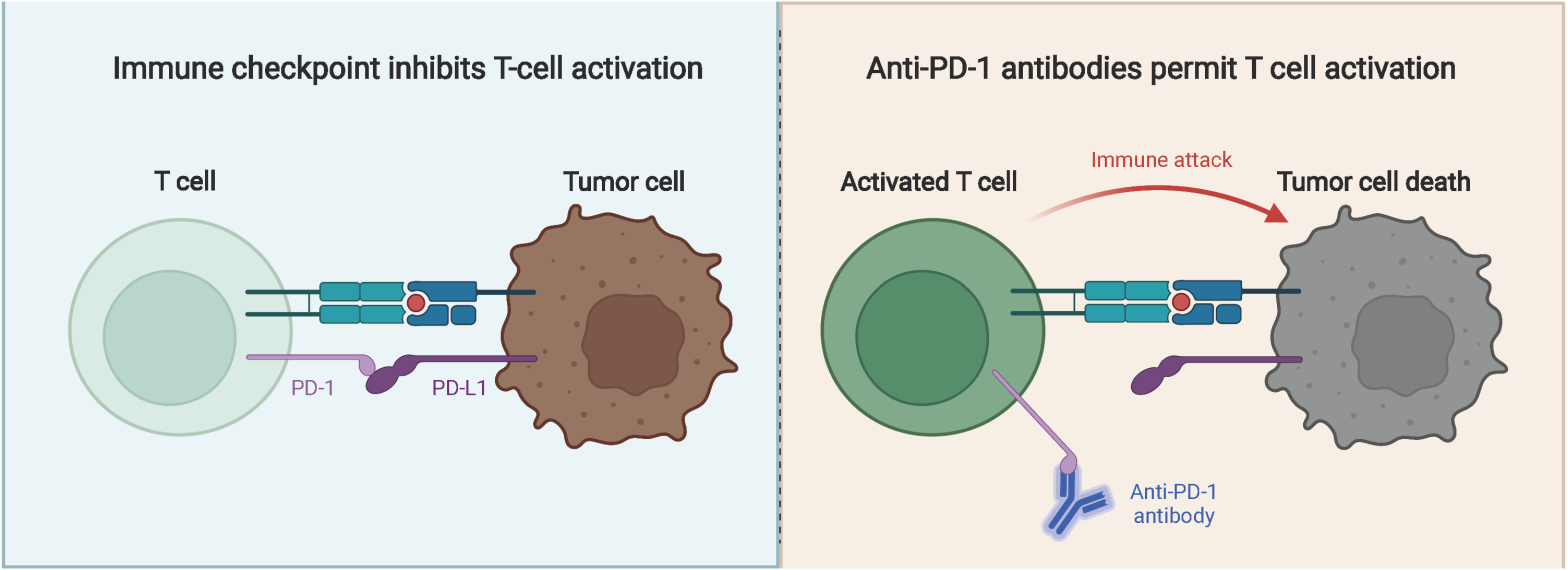
PD‐1 inhibitors activate T cells. The binding of PD‐1 on the surface of T cells to PD‐L1 on the surface of tumor cells inhibits the activation of T cells, while the application of PD‐1 inhibitors promotes the attack of T cells on tumor cells.

Under normal physiological conditions, PD‐1/PD‐L1 signaling pathway activation induces peripheral immune tolerance, which maintains T‐cell immune homeostasis, and prevents immune overactivation‐mediated tissue damage. At the same time, tumor cells use the immunosuppressive function of PD‐1/PD‐L1 to evade host immune surveillance and produce tumor growth‐promoting effects (Figure 3). Given the complex nature of the tumor immune microenvironment, improvements are still needed in molecules or signaling pathways targeting TME for immunotherapy to be effective. The main barriers to the effectiveness of immunotherapy currently include inadequate responses of the host immune system to tumor antigens, low infiltration of immune cells in solid tumors, and formation of immunosuppressive TME.^86^, ^148^

Various immunotherapeutic agents targeting TME have been developed in this stage, however, the combination of diverse immunosuppressive signals makes it challenging for single‐targeted therapeutic regimens to have long‐lasting effects. In the tumor microenvironment, the metabolism between glioma cells and other immune cells promotes the immune escape of glioma cells. Therefore, it is critical to explore the mechanisms by which immune cells obtain sufficient nutrients to maintain their antitumor activity. Although the metabolites of glioma cells can influence the differentiation and function of immune cells, further studies are required to determine whether the intrinsic mechanisms by which metabolites affect immune cells differ in different gliomas. The combination of tumor immunotherapy with metabolic enzyme target inhibitors may maintain the metabolic fitness of TIL cells.^149^ However, glioma cells and immune cells often use the same metabolic pathways for proliferation, so attention needs to be paid to their potential therapeutic toxicity. Selective targeting of tumor cell‐specific metabolic markers may avoid damage to immune cells, significantly promote antitumor immune responses, and reduce the adverse effects of immunotherapy. Additionally, the differential expression of amino acid and nutrient transport proteins between glioma cells and lymphocytes, especially the carriers involved in the interaction, should be considered. These differences make it possible to selectively inhibit tumor cell metabolic pathways.

## AUTHOR CONTRIBUTIONS

Original draft preparation, allocation, and revision: Xu Guo and Rui Sui. Supplement and edition: Haozhe Piao. All authors have read and agreed to the published version of the manuscript.

## FUNDING INFORMATION

This Work is supported by the Fundamental Research Funds for the Central University(2021‐YGJC‐17).

## CONFLICT OF INTEREST STATEMENT

The authors declare no competing interests.

## Data Availability

The data in the current study are available from the corresponding authors upon reasonable request.
